# Acute and Long-Term Effects of Noise Exposure on the Neuronal Spontaneous Activity in Cochlear Nucleus and Inferior Colliculus Brain Slices

**DOI:** 10.1155/2014/909260

**Published:** 2014-07-08

**Authors:** Moritz Gröschel, Jana Ryll, Romy Götze, Arne Ernst, Dietmar Basta

**Affiliations:** Department of Otolaryngology at Unfallkrankenhaus Berlin, Charité Medical School, Warener Straße 7, 12683 Berlin, Germany

## Abstract

Noise exposure leads to an immediate hearing loss and is followed by a long-lasting permanent threshold shift, accompanied by changes of cellular properties within the central auditory pathway. Electrophysiological recordings have demonstrated an upregulation of spontaneous neuronal activity. It is still discussed if the observed effects are related to changes of peripheral input or evoked within the central auditory system. The present study should describe the intrinsic temporal patterns of single-unit activity upon noise-induced hearing loss of the dorsal and ventral cochlear nucleus (DCN and VCN) and the inferior colliculus (IC) in adult mouse brain slices. Recordings showed a slight, but significant, elevation in spontaneous firing rates in DCN and VCN immediately after noise trauma, whereas no differences were found in IC. One week postexposure, neuronal responses remained unchanged compared to controls. At 14 days after noise trauma, intrinsic long-term hyperactivity in brain slices of the DCN and the IC was detected for the first time. Therefore, increase in spontaneous activity seems to develop within the period of two weeks, but not before day 7. The results give insight into the complex temporal neurophysiological alterations after noise trauma, leading to a better understanding of central mechanisms in noise-induced hearing loss.

## 1. Introduction

Long-lasting noise exposure at high intensities leads to an immediate posttraumatic temporary shift of hearing thresholds (TTS) and is followed by a long-lasting permanent threshold shift (PTS) if sensory tissue is damaged to a large extent at high sound intensities [1, 2]. It has already been shown that PTS is accompanied by dramatic changes of cellular properties within the central auditory pathway, such as neuronal shrinkage, axonal and synaptic degeneration [3–6], changes in synaptic activity [7, 8], and a decrease in neuronal cell density [9, 10].

Electrophysiological research has shown that spontaneous neuronal activity and compound action potentials are decreased in the auditory nerve after noise exposure [11, 12]. Similar findings have been reported within the first days postexposure in the central structure of the dorsal cochlear nucleus [13, 14]. However, long-lasting effects of noise trauma are an upregulation of spontaneous firing rates in several structures of the central auditory pathway [14–16] and an increase in excitability, whereby excitatory thresholds are elevated [17]. These changes seem to be based upon both a reduction of GABAergic inhibition and an enhancement of excitation within the impaired structures [18, 19].

It is still a matter of debate if the observed effects are related to the noise-induced changes of afferent peripheral input or directly evoked by an overstimulation of the entire auditory pathway. It seems that central auditory structures contribute to the development of an acute auditory threshold shift since it has been shown that some central effects of noise could be short-term only [10, 20]. Furtherly, clinical sequalae of noise exposure, such as tinnitus and hyperacusis, are frequently present after the hearing loss has recovered which cannot be fully explained with cochlear pathology [21]. These discrepancies are possibly related to additional, central mechanisms involved in the generation of noise-induced hearing loss (NIHL). To date, only a few studies focused on the time course of the electrophysiological changes after noise trauma. However,* in vivo* findings indicate that hyperactivity in the central auditory system develops during the first days after trauma [22], whereby the DCN seems to play a key role in maintaining the increased neuronal firing throughout the ascending pathway [23, 24].

Electrophysiological changes in the central auditory system have been commonly studied* in vivo* (anaesthetized animals). Thereby, it is hardly possible to differentiate if changes in central auditory processing rely on altered central auditory structures under observation or if they were influenced by interacting ascending and descending neuronal projections. By using electrophysiological recording techniques in brain slices, intrinsic cellular changes of different levels of central auditory processing could be evidenced. With this methodology, single brain structures disconnected from peripheral and central afferent and efferent input can be investigated in detail.

The aim of the present study was therefore to describe the temporal patterns of spontaneous, neuronal single-unit activity upon noise-induced hearing loss in different key structures of the lower central auditory pathway. This experimental procedure allows distinguishing between acute and long-term changes in the dorsal and ventral cochlear nucleus as well as the inferior colliculus.

## 2. Methods

### 2.1. Noise Exposure

57 normal hearing adult mice (NMRI-strain, age 1 to 3 months) of both sexes were investigated. The experimental protocol was approved by the governmental commission for animal studies (LaGeSo Berlin, approval number: G 0416/10). Experiments were carried out in accordance with the EU Directive 2010/63/EU on the protection of animals used for scientific purposes. All efforts were made to minimize pain or discomfort.

The animals were anaesthetized for 3 h with ketamine/xylazine (60 mg/kg ketamine, 6 mg/kg xylazine). 32 mice were exposed to noise during the anaesthesia in a sound-proof chamber (0.8 m × 0.8 m × 0.8 m, minimal attenuation 60 dB SPL) with a broad-band white noise (5–20 kHz) at 115 dB SPL. Noise was delivered binaurally by high tone loudspeakers (HTC 11.19, Visaton, Haan, Germany) placed above the animal's head. Speakers were connected with an audio amplifier (Tangent AMP-50, Aulum, Denmark) and a DVD Player (DK DVD-438, Ratingen, Germany). Sound-pressure level (SPL) was calibrated by using a sound level meter (Voltcraft 329, Hirschau, Germany) placed close to the animal's ear. Anaesthesia was controlled by a video camera inside of the lighted chamber. Body temperature was maintained at a constant level of 37°C. Mice were investigated immediately after the noise exposure (acute group, *n* = 9) or were kept in their cages for one (7-day group, *n* = 17) or two (14-day group, *n* = 6) weeks to be investigated seven or fourteen days later. Unexposed animals were used as controls (control group, *n* = 25).

### 2.2. Electrophysiological Recordings

**Figure 1:**
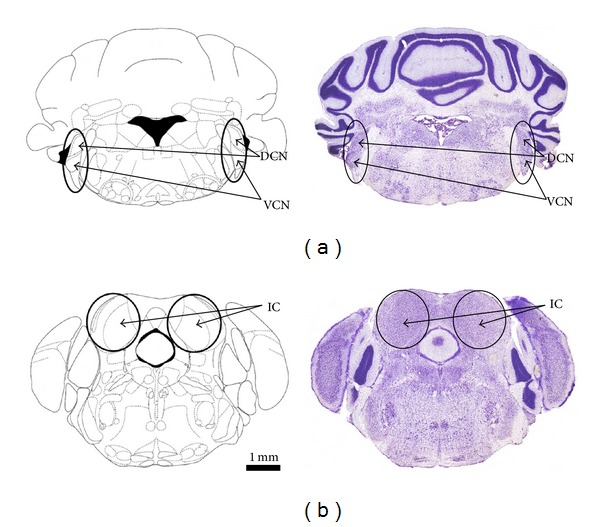
Transversal schematic diagrams (left) and corresponding histological sections (Nissl-stained 40 *μ*m brain slice preparations, right) from the mouse brain including the investigated structures of the cochlear nucleus (black labelled area in (a)) in the brainstem with its dorsal and ventral subdivision (DCN and VCN, resp.) and the inferior colliculus (IC, black labelled area in (b)) in the midbrain. Layers correspond to the prepared brain slice sections. Pictures were taken and modified from the mouse brain atlas by Paxinos and Franklin [25].

Extracellular single-unit recordings were performed in acute living brain slices to investigate the spontaneous firing rates at different stages after noise trauma (acute group, *n* = 9; 7-day group, *n* = 17; 14-day group, *n* = 6) compared to unexposed controls (*n* = 25). Therefore, the animal (only one animal was investigated at each experimental day) was decapitated and the brain was carefully taken out. Using a vibrating microtome (Model 1000 plus, Vibratome, St. Louis, Missouri, USA), 300 *μ*m thick frontal slices (including CN or IC, resp.) were microdissected. After 2 h of incubation in carbogenized (95% O_2_–5% CO_2_) artificial cerebrospinal fluid (cACSF) at 35°C under submerged conditions, one slice was transferred to a submerged-type recording chamber. The recording chamber was continuously perfused (2.5 mL/min) with warm (36°C) cACSF. The cACSF contained the following concentrations (in mM): 124 NaCl, 3 KCl, 1.25 NaH_2_PO_4_, 1.8 MgSO_4_, 1.6 CaCl_2_, 10 glucose, and 26 NaHCO_3_. The temperature of the bath solution was measured with a thermistor probe in the recording chamber and regulated within a small range by a temperature controller (SCTC-20E, npi-electronics, London, UK). Action potentials from spontaneously active neurons within the dorsal and ventral cochlear nucleus (DCN and VCN, resp.) and the inferior colliculus (IC) (localized using the stereotaxic brain atlas from Paxinos and Franklin [25], Figure 1) were recorded with glass electrodes (GB120-F10, Science Products, Hofheim, Germany). The electrodes were pulled on a P97 horizontal puller (Sutter Instruments, Novato, CA, USA) and back-filled with a sodium chloride solution (154 mM). The resulting electrode resistance was approximately 2 MΩ. The signal was amplified (10,000x) and filtered (0.3–20 kHz band pass) (Model 1800, A-M Systems Inc., Sequim, Washington, USA), visualized on an oscilloscope, digitized by a 1401 Plus interface (Cambridge Electronic Design Ltd., Cambridge, UK), and stored in the Spike2 software format (Cambridge Electronic Design Ltd., Cambridge, UK). Spikes were detected offline by using the Spike2 software. After establishing a stable recording, neuronal spontaneous activity was measured for 3 minutes.

### 2.3. Statistical Procedures

The resulting mean neuronal spontaneous activities (events per second) of the experimental groups were compared with the controls for each brain region by the *U*-test as the data were not normally distributed. Moreover, each investigated structure was topographically subdivided into a high- and low-frequency area. Data of these subdivisions have been compared within each structure as well as between frequency-related substructures of different experimental groups using *U*-test (not normally distributed data) or *t*-test (normally distributed data). Data distribution was tested by applying the Kolmogoroff-Smirnoff test. The software SPSS (IBM SPSS Statistics Version 20, IBM Corp., Armonk, New York, USA) was used for all statistical analyses. The level of significance was *P* < 0.05. A Bonferroni correction was applied for multiple comparisons.

## 3. Results

### 3.1. Cochlear Nucleus

**Figure 2:**
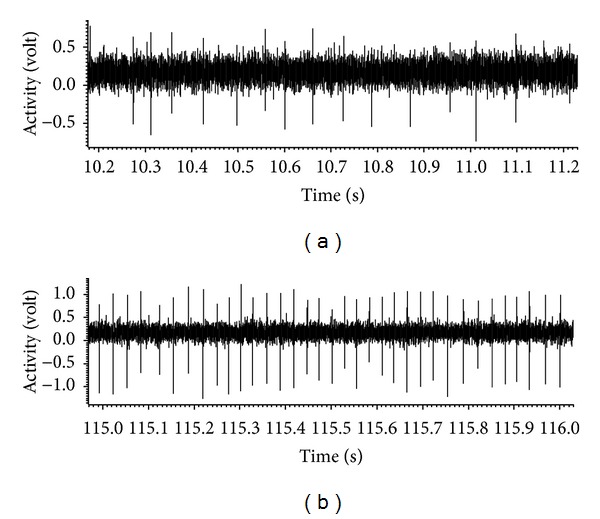
Example of filtered and amplified spike trains of DCN neurons from a control (a) and a 14-day group (b) animal, recorded with the spike2 software. Single units were classified using the template matching function of the spike2 software and mean spontaneous activity was calculated accordingly from the original recordings. Each diagram represents a one-second frame out of a 3-minute recording with the time in seconds on the *x*-axis and the activity in volt on the *y*-axis. The higher spike rate of the 14-day group neuron indicates the increase in spontaneous firing in the noise-exposed group two weeks after trauma.

The extracellular electrophysiological recordings in brain slices showed significant changes in spontaneous activity immediately as well as two weeks after noise exposure compared to controls. Interestingly, spontaneous neuronal firing rates did not show significant differences at day 7 after trauma. An example of two DCN neurons spike trains from a control and a 14-day group animal is given in Figure 2. An increase in spontaneous firing in the noise-exposed group two weeks after trauma is represented by the higher spike rate of the 14-day group neuron (Figure 2).

**Figure 3:**
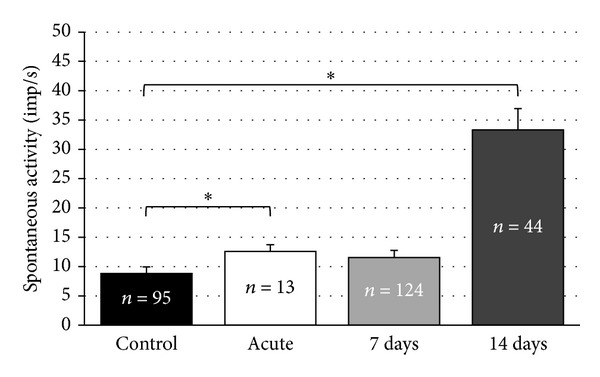
Spontaneous activities (mean ± S.E.) of recorded neurons in the dorsal cochlear nucleus (DCN) of the control and noise-exposed groups. Numbers of recorded units for each subgroup are inserted inside of the columns. Asterisks indicate significant differences.

**Figure 4:**
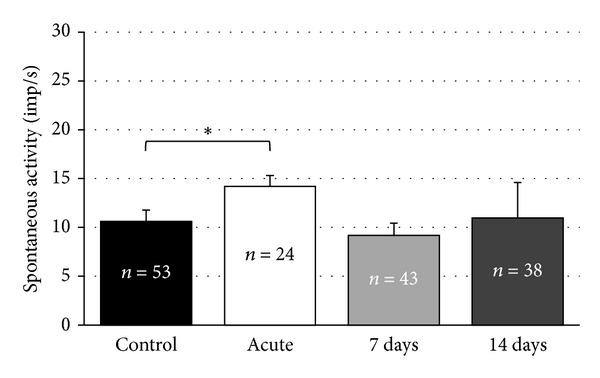
Spontaneous activities (mean ± S.E.) of recorded neurons in the ventral cochlear nucleus (VCN) of the control and noise-exposed groups. Numbers of recorded units for each subgroup are inserted inside of the columns. Asterisks indicate significant differences.

The mean rate of action potentials in the recorded DCN units was increased significantly from 8.8 ± 1.4 imp/s (95 recorded neurons) in the control group to 12.6 ± 1.8 imp/s (13 recorded neurons) acutely after noise exposure (*P* = 0.002; Figure 3). A similar observation has been made in the VCN of the acute group, where the mean spontaneous firing rate was 14.2 ± 1.8 imp/s (24 recorded neurons) compared to 10.6 ± 2.1 imp/s (53 recorded neurons) in the control group (*P* = 0.001; Figure 4). Changes in spontaneous neuronal firing rates were not significantly different in the 7-day group when compared to controls. This holds true for the DCN (7 days: 11.5 ± 1.6 imp/s (124 recorded neurons), *P* = 0.131) and the VCN (7 days: 9.2 ± 2.1 imp/s (43 recorded neurons), *P* = 0.979).

When analyzing the 14-day group data, it became evident that significant differences were present in the DCN, but not in the VCN compared to controls. In the DCN, mean spontaneous activity was raised to 33.3 ± 6.3 imp/s (44 recorded neurons, *P* = 0.001; Figure 3) and was not statistically significantly elevated in the VCN (11.0 ± 1.2 imp/s (38 recorded neurons), *P* = 0.019; Figure 4).

### 3.2. Inferior Colliculus

**Figure 5:**
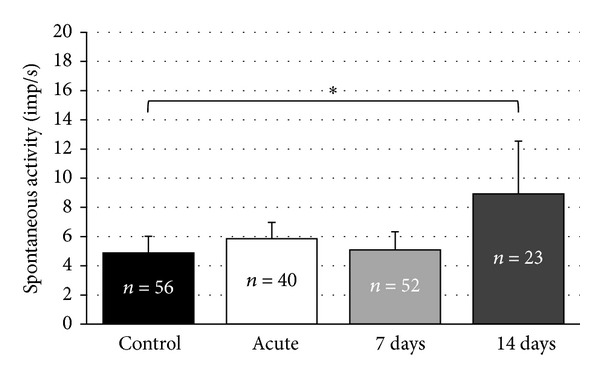
Spontaneous activities (mean ± S.E.) of recorded neurons in the inferior colliculus (IC) of the control and noise-exposed groups. Numbers of recorded units for each subgroup are inserted inside of the columns. Asterisks indicate significant differences.

The spontaneous activity in the IC immediately after the noise trauma was not significantly different to controls (control group: 4.9 ± 0.4 (56 recorded neurons), acute group: 5.9 ± 0.5 (40 recorded neurons), *P* = 0.55; Figure 5).

Changes in spontaneous neuronal firing rates were also not significantly different in the 7-day group when compared to control data (5.1 ± 0.6 imp/s (52 recorded neurons), *P* = 0.3; Figure 5).

Two weeks after the noise exposure, the spontaneous activity was significantly higher than in controls (8.9 ± 1.3 imp/s (23 recorded neurons), *P* = 0.014; Figure 5).

The original recordings did not show large differences in neuronal firing patterns at different time points after noise exposure. As indicated by the sample recordings (Figure 2), neuronal firing in general was quite regular without any certain changes in firing characteristics between the experimental or control groups.

Statistical analysis of tonotopically related spontaneous activity did not show any significant effects. Neither within-group comparison between high- and low-frequency areas in each structure, nor comparison of matched subdivisions for each structure between experimental and control groups showed any frequency-related differences in changes of spontaneous activity. Thus, neuronal firing rates are supposed to show an equal distribution across the investigated auditory structures.

## 4. Discussion

The results of the present study demonstrate that acoustic overstimulation changes physiological properties of neurons within the lower central auditory pathway with a distinct temporal pattern. With our specific experimental design, we showed that the increases in neuronal firing rates are genuine, intrinsic cellular changes in isolated central auditory structures.

### 4.1. Hearing Thresholds

**Figure 6:**
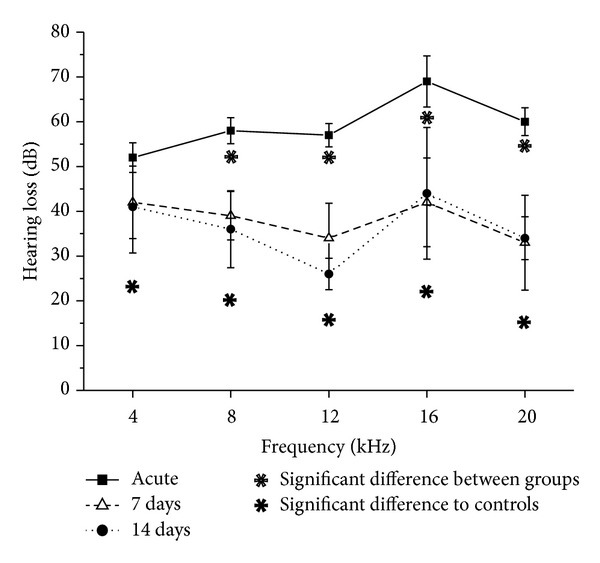
Threshold shift in dB SPL (mean ± S.E.) of the auditory brainstem response (ABR) at different frequencies of noise-exposed mice (acute group: filled squares; 7-day group: open triangles; 14-day group: filled circles). Filled asterisks show significant differences between treatment groups and controls. Open asterisks indicate significant recovery of auditory thresholds between the acute and 7-day group (from [26]).

Previous studies showed that the applied noise paradigm leads to a significant elevation in auditory thresholds in all experimental groups with the highest shift in the acute group, followed by a significant recovery within one week (Figure 6, from [26]). However, hearing loss was still significantly increased after 7 days compared to controls and showed no further changes until day 14, indicating that a permanent threshold shift (PTS) has developed.

Several studies have already reported that short-term processes within the cochlea (reversible and irreversible damage of sensory tissue) contribute to the acute threshold shift. A strong peripheral excitation by glutamate release reduces the energy and transmitter availability [1] and thereby inhibits further excitability. Similar mechanisms in central auditory structures might be responsible to some extent for the generation of sudden hearing loss.

### 4.2. Acute Effects

The above-mentioned idea is strongly supported by the present finding of a slight, but significant, elevation in spontaneous firing rate in the acute group at the first level of central auditory processing, that is, the cochlear nuclei. No differences were found in hierarchically higher structures. These experimental findings suggest that the applied noise trauma exerts a direct influence on the intrinsic, cellular network activity of the CN which in turn triggers several other pathophysiological events. This is also supported by a recent study of our group indicating a large increase in calcium-dependent activity* in vivo* immediately after acoustic overexposure [26].

An acute increase of peripheral activation of inner hair cells and spiral ganglion neurons [1] could induce a strong glutamate release at synapses between auditory nerve fibres and CN neurons, particularly at highly calcium-permeable, fast-transmitting AMPA receptors at the endbulb of Held's synapses. This leads to a fast and powerful long-lasting excitation [27]. These mechanisms might strongly contribute to acute hearing loss and result in short-term plastic changes [28], which is indicated by maintained excitatory transmitter release even after slice preparation. An immediate posttraumatic activity increase was also reported for the CN* in vivo* [29]. This overexcitation could induce neurodegenerative mechanisms such as apoptosis or even necrosis [30] as supported by recent findings on acute cell death in the CN after noise exposure [10, 31]. An acute elevation in spontaneous activity in anaesthetized animals was also present in higher auditory structures, namely, the IC [16, 32]. This effect was missing in our results and argues that IC hyperactivity at this time point is not based upon sustained physiological changes within this nucleus, but it is dependent on afferent (and perhaps efferent) connectivity. The absence of activity changes could also arise from protective mechanisms to reduce the direct impact on higher structures. Traumatic injury could be prevented by activation of inhibitory interneurons in the CN suppressing ascending neural transmission [33]. Moreover, rapid reduction in postsynaptic AMPA-receptor densities could avoid excitotoxicity. A similar mechanism was demonstrated in spiral ganglion neurons of the auditory nerve [34].

### 4.3. Late Effects

One week postexposure, a strong permanent hearing loss was established due to irreversible peripheral and central damage. In the cochlea, a loss of sensory tissue (hair cells and spiral ganglion neurons) has been observed [35, 36] resulting in reduced stimulus conduction to the auditory nuclei. Moreover, cell densities of involved brain areas are largely decreased at this time point due to apoptotic mechanisms [9, 10, 31]. The PTS findings at one week were confirmed by the measurements of the 14-day group.

Single-unit responses remained unchanged in all CN subdivisions as well as in the IC one week postexposure. However, when looking at the data of the animals investigated at 14 days after noise exposure, it turned out that intrinsic long-term hyperactivity in brain slices of central auditory structures was detected for the first time in the present study. Therefore, increase in neuronal spontaneous firing seems to develop within the period of two weeks, but not before day 7 after trauma. In general, mean spontaneous activity was much less expressed in the IC compared to the hierarchically lower CN. This finding is in line with other studies investigating neuronal spontaneous firing in these structures before and after noise exposure [23]. Previous* in vivo* studies pointed out those long-lasting hyperactive disorders in central auditory structures after noise exposure, particularly within the lower auditory pathway, namely, the DCN and IC [14, 16, 37, 38]. These changes were shown to be induced within the first week after acoustic trauma and to gradually be increased in magnitude during the following days and months [22]. The DCN as well as an intact cochlea seems to be important for the maintenance of IC hyperactivity, as shown by* in vivo* lesioning studies [24, 37]. However, the intrinsic neuronal properties in both DCN and IC seem to be changed over time and are even present after isolation of the particular structures. It can be hypothesized that other neural connections, for example, descending projections from the auditory cortex, still have a significant impact on the reorganization of neurotransmission after noise trauma in the live animal [39–41]. Nevertheless, the DCN plays a fundamental role as a relay for transmitting hyperactivity towards the IC [23], though our data suggest that the effects as reported for IC cells are not simply a passive response. This idea is also supported by Robertson et al. [42] who showed in guinea pigs that IC hyperactivity becomes independent of afferent neuronal input over time. Although the observed effects occur later compared to our findings, the involved mechanisms might be similar.

Brain slice recordings after a pure-tone noise exposure indicate that hyperactive disorders are partially caused by a lack of inhibition in the affected neurons [18, 43]. These studies showed reduced firing rates in CN and IC neurons of animals with noise-induced hearing loss which could be due to an increased postsynaptic GABA-receptor density. An increase in inhibitory glycine activity further contributes to the effect [44]. This causes a decline in spiking activity after slice preparation by removing the neuronal input to the relating auditory structures [45]. Differences to our present findings could be caused by the broad-band noise exposure in this study, followed by more widespread tissue damage with compensatory neuroplasticity, compared to narrow-band or pure-tone sounds. This is confirmed by the equal distribution of neurons with specific firing rates across the dimensions of the investigated structures, accompanied by a significant overall shift in auditory thresholds in the noise-exposed animals across the entire tested frequency range. A broad-band noise trauma might therefore induce a different redistribution of posttraumatic excitatory and inhibitory neuronal properties, such as transmitter release, receptor densities, and synaptic strengths. Further, earlier studies were able to show that hyperactive disorders due to noise-induced hearing loss are often related to the frequency area of the detected hearing loss [16, 46]. Thus, the absence of location- (frequency-) specific hyperactivity in our data is possibly related to the induced extensive hearing loss, which has been detected in the present study.

Particularly in the DCN, an upregulation of cholinergic and glutamatergic neuronal input also seems to play an important role in generating hyperactivity [19, 47–49]. The shift in the balance of inhibition and excitation due to synaptic plasticity as compensatory response to neuronal deafferentiation seems to be the trigger of central hyperactivity [50]. The involved mechanisms could range from axonal sprouting and regrowth of synaptic connectivity in response to a loss of input [51–54] or homeostatic changes shifting the strengths of existing synapses due to input alterations [55–57]. Neuronal hyperactivity might also develop in response to central degeneration to account for the deterioration of neural tissue. Recent studies of our group have demonstrated that cell death is particularly present within the first week after this noise trauma paradigm [10, 31]. Neuroplasticity might lead to profound changes in synaptic transmission during this development, but the present data support the assumption that spontaneous hyperactivity seems to be driven by an intact neural network at this time point. Although anatomical changes within the second week after noise trauma have not been investigated until now, it could be hypothesized from calcium activity monitoring that neuroplasticity and possibly neurodegeneration are largely reduced [26], and hyperactivity as a pathophysiological disorder might already be manifested within the neuronal structures of the lower central auditory pathway at this time.

## 5. Conclusion

The aim of the present study was to describe the temporal patterns of spontaneous, neuronal single-unit activity upon noise-induced hearing loss in brain slices of the dorsal and ventral cochlear nucleus as well as the inferior colliculus. This experimental procedure allows measuring the intrinsic acute and long-term activity changes in structures of the lower auditory pathway isolated from afferent or efferent neuronal input. An increased spontaneous activity seems to develop within two weeks, but not before day 7 after acoustic trauma. Altogether, the present study provides a deeper insight into the complex alterations in neuronal physiological properties within the adult central auditory system after a single noise trauma. The results should contribute to a better understanding of central mechanisms in acute and permanent noise-induced hearing loss.
